# Unani Tibb practitioners’ perceptions and attitudes towards spirituality and spiritual care in Unani Tibb practice in South Africa

**DOI:** 10.1186/s12906-023-04002-y

**Published:** 2023-06-09

**Authors:** Mujeeb Hoosen, Nicolette Vanessa Roman, Thuli Godfrey Mthembu, Mursaleen Naseer

**Affiliations:** 1 School of Natural Medicine, Faculty of Community and Health Sciences, The University of the Western Cape, Bellville, South Africa; 2 Centre for Interdisciplinary Studies of Children, Family and Society, Faculty of Community and Health Sciences, The University of the Western Cape, Bellville, South Africa; 3 Department of Occupational Therapy, Faculty of Community and Health Sciences, University of the Western Cape, Bellville, South Africa; 4 Department of Moalejat, Ajmal Khan Tibbiya College, Faculty of Unani Medicine, Aligarh Muslim University, Aligarh, India

**Keywords:** Unani tibb, Perception, Attitude, Spirituality, Spiritual care, Clinical practice, Practitioners, South africa

## Abstract

**Background:**

Unani Tibb is an Arabic term which means Greek medicine. It is an ancient holistic medical system based on the healing theories of Hippocrates, Galen and Ibn Sina (Avicenna). Despite this, spirituality and spiritual care practices are deficient in the clinical setting.

**Methods:**

This cross-sectional descriptive study was used to describe Unani Tibb practitioners’ perceptions and attitudes regarding spirituality and spiritual care in South Africa. A demographic form, Spiritual Care-Giving Scale, Spiritual and Spiritual Care Rating Scale and Spirituality in Unani Tibb Scale were used to collect data.

**Results:**

A response rate of 64.7% (*n* = 44 out of 68) was achieved. Positive perceptions and attitudes regarding spirituality and spiritual care were recorded for Unani Tibb practitioners. The spiritual needs of their patients were considered vital towards enhancing the Unani Tibb treatment approach. Spirituality and spiritual care were regarded as fundamental to Unani Tibb therapy. However, most practitioners agreed that adequate training in spirituality and spiritual care was lacking and future training initiatives were imperative for Unani Tibb clinical practice in South Africa.

**Conclusion:**

The findings of this study recommends further research in this field by means of qualitative and mixed methods approaches to provide a deeper understanding to this phenomenon. Clear guidelines on spirituality and spiritual care for Unani Tibb clinical practice are essential to ensure the integrity of the holistic approach required by the profession.

## Background

Unani Tibb, also known as Unani medicine or Tibb, is one of the oldest and most accepted forms of complementary and alternative medicine (CAM) practiced in 82 of the World Health Organization (WHO) member states, which includes South Africa [1, 2]. Unani Tibb embraces the science of medicine and the art of healing [2]. Holism is the central component of the Unani Tibb philosophy [3]. This holistic approach integrates the multidimensional components of life which include spirituality [3–5]. WHO highlights the importance of spirituality and CAM in the health and well-being of all populations globally [1, 2, 6].

Spirituality and CAM are interconnected in several ways [7–11]. Worldwide, CAM practitioners and consumers have shown growing interest in prescribed and self-use spiritual therapies [12]. All CAM philosophies acknowledge the mind, body and spirit relationship. It is a fundamental component of CAM, often discussed in the context of holistic care [10, 13–15]. Several studies reported that spirituality was the strongest predictor for CAM use amongst most patients affected by chronic illnesses [7–10, 12, 13, 16, 17]. Healthcare practitioners, who provide spiritual care to their patients contribute significantly to improve their overall well-being [5, 8, 10–12, 16–21].

Consequently, the importance of spirituality and spiritual care has received attention in most healthcare professions [10, 13–15, 22]. However, this is not the case for CAM professions such as Unani Tibb [10, 13–15]. Unani Tibb is a modality of CAM practiced in South Africa [15, 23]. CAM is a prominent component of the healthcare delivery in South Africa with an estimated 70–80% of the South African population seeking some form of CAM treatment [15, 23]. Patients associate spirituality within the CAM domain [12, 14, 16, 17] and expect spiritual care from Unani Tibb practitioners [3, 14, 15, 24, 25].

In Unani Tibb illnesses are viewed as multifactorial in nature, consisting of physical, emotional, mental and spiritual components [1–3, 14, 15, 24, 25]. The human being is made up of body and spirit and a balanced life can only be achieved when proper attention is given to both physical and spiritual functions [3, 14, 15, 24–26]. Consequently, Unani Tibb practitioners are expected to place a considerable amount of emphasis on the patient’s spiritual and social life [3, 14, 15, 24, 25]. However, this does not seem to be the case in the South African context. The Unani Tibb treatment approach should go beyond the patient’s pathology and psychology. The practitioner should also focus on relevant aspects relating to the family, community and spirituality [3, 14, 15, 24]. Spirituality and spiritual care are core components to the Unani Tibb philosophy; however, these concepts are absent in clinical practice. The theory–practice gap with regard to spirituality and spiritual care has also been reported in other health professions literature [5, 10, 13–15, 27, 28].

Research on spirituality and spiritual care in Unani Tibb is extremely rare [14, 15, 26]. In one study, Ahmad (2015) reports that Unani Tibb students considered spirituality important to their training and clinical practice. This study reported on a significant relationship between the spiritual personality of students and the level of empathy they expressed to their patients. In a multi-site exploratory study, Unani Tibb and other CAM practitioners agreed that spirituality was a focus area for their patients during illness [10, 14, 15, 26]. These studies reported that Unani Tibb practitioners supported the inclusion of spirituality and spiritual care into clinical practice [10, 14, 15, 26]. However, there seems to be uncertainties regarding the role of spirituality and spiritual care in Unani Tibb clinical practice which requires further investigation in the South African context [14, 15].

## Methods

### Study design and sample

A quantitative cross-sectional descriptive design was used for this study to describe Unani Tibb practitioners’ perceptions and attitudes regarding spirituality and spiritual care in clinical practice. A non-probability convenience sampling method was used to select all registered Unani Tibb practitioners (*n* = 68) to participate in the study. A total number of 68 online self-report questionnaires were emailed as a link of Google form together with an information sheet explaining the purpose of the study. A response rate of 44 of 68 (64.7%) practitioners participated in the study.

This study was approved by the Biomedical Research Ethics Committee and Higher Degrees Committee (ethical clearance: BM20/2/7) at the University of the Western Cape. Participation in the study was voluntary for all Unani Tibb practitioners. The participants were provided with an information sheet explaining the purpose of the research study requesting their participation and assuring them confidentiality. All data were de-identified and kept in a safe place only accessible to the researchers. Informed consent was received from all participants in the study. Participants were given the right and opportunity to withdraw from the study at any time, without any repercussions. All online submitted questionnaires were protected with a password for Google Drive.

### Data collection instruments

Data were collected by means of an online self-reported questionnaire adapted from previous studies on spirituality and spiritual care [22, 27–34].

### Participant demographic characteristics

The demographic information regarding age, gender, marital status and religion was obtained in this study. Marital status was divided into single, married, divorced and separated.

### Spiritual care-giving scale (SCGS) [30–33]

The SCGS comprises five factors with a six-point Likert scale, which was developed and tested to be valid and reliable (a = 0.96). The five factors include: (1) Attributes for Spiritual Care, (2) Spirituality Perspective, (3) Defining Spiritual Care, (4) Attitudes to Spiritual Care and (5) Spiritual Care Values. In a previous study the Cronbach’s alpha value for this scale was 0.94 [34]. Akin et al. reported a Cronbach’s alpha value of 0.96 [35]. In the current study, the Cronbach’s alpha value for this scale was 0.91. Permission to use the 35-item SCGS questionnaire was granted by Dr Lya Hwa Tiew on 24 April 2019.

### Spirituality and spiritual care rating scale (SSCRS) [22]

The SSCRS was designed to explore individual nurses’ beliefs and values. This scale consists of the following four factors: (1) Spirituality; (2) Spiritual Care; (3) Religiosity; and (4) Personalised Care. The 17-item scale uses a five-point Likert scale response option. This instrument demonstrated modest internal consistency with a Cronbach’s alpha of 0.64. In another study the Cronbach’s alpha value for this scale was 0.76 [34]. The Cronbach’s alpha of the scale was 0.69 in this study. Permission to use the 17-item SSCRS questionnaire was granted by Prof. Wilfred McSherry on 3 May 2019.

### Spirituality Unani Tibb scale (SUTS)

The SUTS was adapted from the Spirituality in Occupational Therapy (SOT) questionnaire which is one of the reliable tools previously used to measure spirituality in occupational therapy [27]. The SOT was designed specifically to examine occupational therapists’ self-reported perceptions regarding: (1) spirituality in the scope of practice following its addition in the theoretical framework, (2) formal education and training in spirituality, (3) the need for future educational opportunities and training to address spirituality and (4) awareness of assessments and evaluations in occupational therapy that incorporate clients’ spirituality.

The 20-item scale uses a five-point Likert-type scale response option. In a previous study the Cronbach’s alpha value for this scale was 0.87 [34]. In the present study, the Cronbach’s alpha value for this scale was 0.78. Permission to use the 20-item SOTS questionnaire was granted by Dr Douglas N. Morris on 24 April 2019.

### Procedure

The Directors of the South African Tibb Association (SATA) were consulted to request permission to conduct this study and gain access to the Unani Tibb practitioners’ mailing list. SATA is a national body representing the Unani Tibb profession in healthcare delivery and education in South Africa. Unani Tibb practitioners were given information about the study by means of email. The online self-questionnaire was sent as a link for Google forms to the practitioners.

The practitioners completed the questionnaire online. Their responses were automatically calculated online, then exported to Microsoft Excel 2010 to create a compatible data set for statistical analysis. Statistical Analysis Descriptive data analysis was performed using the Statistical Package for the Social Sciences (SPSS) software 20.0 (SPSS, Inc, Chicago, IL, USA).

The data were cleaned and coded in preparation for analysis. Descriptive statistics were used to characterise demographics with number (n) and percentages (%). For the variables from the scales (SCGS, SSCRS and SUTS), proportions, mean scores and standard deviations are reported. A higher score of the mean in the scales indicated a higher level of agreement and a more positive perception and attitude about spirituality and spiritual care for Unani Tibb practitioners.

### Validity and reliability

The reliability of the SCGS, SSCRS and SOT has been previously established. The SCGS was developed and tested to be valid and reliable (a = 0.96) [29]. The 17-item SSCRS demonstrated a reasonable level of internal consistency reliability, having a Cronbach’s alpha coefficient of 0.64. Mthembu et al. [34] reported on the validity and reliability (a = 0.70) in a previous study.

In this study, stability and consistency were both assessed for the reliability of the questionnaire. In a previous study, Mthembu et al. [34] reported on the validity and reliability (a = 0.87) of SOT. Validity refers to the degree to which what is being measured is what the researchers intended [30–33]. For the purpose of the study, validity was enhanced through the use of previously validated questionnaires. Face and content validity of the instrument were considered in this study [34].

## Results

### Participant demographic characteristics

A final sample of 44 Unani Tibb practitioners participated in the study. The response rate was 64%, and practitioners were reminded several times about the study through email. Table 1 provides a summary of the demographic characteristics of the participants.

**Table 1:** Participant demographic characteristics (44)

*Variables*	*N* = 44	%
**Gender**		
** Male**	9	20.5
** Female**	35	79.5
**Language**		
** English**	36	81.8
** isiXhosa**	6	13.6
** Other**	2	4.5
**Marital Status**		
** Single**	15	34.1
** Married**	29	65.9
**Religion**		
** Yes**	43	97.7
** No**	1	2.3

The majority of the participants in this study were females (*n* = 35; 79.5%). The mean age of the study sample was 38.4 years with a range of 23–66 years. The majority of practitioners were English speaking (*n* = 36; 81.8%). In this study, 65.9% of Unani Tibb practitioners were married and belonged to a religion (97.7%).

### Unani Tibb practitioners’ religion, spirituality and beliefs

In this study, two-thirds of participants (66%, *n* = 29) reported Islam as a religion, 27% (*n* = 12) reported Christianity and 5% (*n* = 2) reported other. The results of this study also show that 91% (*n* = 40) of the participants attended and engaged in organised religious group activities. For some practitioners (34%, *n* = 15) these religious events were attended more than once a week, 27% (*n* = 12) reported attending these events once a week, 22% (*n* = 10) attended occasionally and once a year and 6.8% (n = 3) attended once a month. One participant (*n* = 1; 2.3%) reported involvement in non-religious activities such as meditation, scriptural study group and prayer.

Most Unani Tibb practitioners (*n* = 26; 59.1%) indicated that they perceived themselves as spiritual compared to 40.9% (*n* = 18) who revealed that they perceived themselves as religious.

### Responses to the SCGS

Table 2 provides a summary of the results for mean scores and standard deviations of spirituality and spiritual care by Unani Tibb practitioners. Item 33 had the lowest mean ‘The ability to provide spiritual care develops through experience’ (M = 2.39, SD = 0.900), and the highest mean was for item 37, ‘Spirituality helps when facing life’s difficulties and problems’ (M = 5.55; SD = 0.627). In this study, mean values were computed for each factor. The mean value for factor 1 was 5.28 (SD = 0.502); 5.20 (SD = 0.867) for factor 2; 5.14 (SD = 0.776) for factor 3; 5.05 (SD = 0.578) for factor 4 and 5.08 (SD = 0.653) for factor 5.

**Table 2:** Scores on the SCGC (*n* = 44)

Variables	*M*	*SD*
**Factor 1—Attributes for Spiritual Care**	5.28	0.502
**Q27. Spiritual care should consider what patients think about spirituality**	5.20	0.851
**Q28. Unani Tibb practitioners who are spiritual aware are more likely to provide spiritual care**	5.32	0.740
**Q29. Spiritual care requires awareness of one's spirituality**	5.45	0.589
**Q33. The ability to provide spiritual care develops through experience**	2.39	0.900
**Q36. Spirituality is influenced by individual’s life experiences**	5.18	0.971
**Q37. Spirituality helps when facing life’s difficulties and problems**	5.55	0.627
**Q38. Spiritual care requires the Unani Tibb practitioner to be empathetic towards the patient**	5.43	0.728
**Q39. A trusting Unani Tibb practitioner-patient relationship is needed to provide spiritual care**	5.09	1.074
**Factor 2—Spirituality Perspective**	5.20	0.867
**Q1. Everyone has spirituality**	4.48	1.422
**Q2. Spirituality is an important aspect of human beings**	5.52	0.902
**Q3. Spirituality is part of a unifying force which enables individuals to be at peace**	5.39	1.017
**Q4. Spirituality is an expression of one’s inner feelings that affect behaviour**	5.14	1.069
**Q5. Spirituality is part of our inner being**	5.30	1.025
**Q6. Spirituality is about finding meaning in the good and bad events of life**	5.05	1.257
**Q7. Spiritual well-being is important for one’s emotional well-being**	5.41	1.106
**Q8. Spirituality drives individuals to search for answers about meaning and purpose in life**	5.36	1.102
**Factor 3—Defining Spiritual Care**	5.14	0.776
**Q14. Spiritual care is a process and not a one-time event or activity**	5.52	0.731
**Q15. Spiritual care is respecting a patient’s religious or personal beliefs**	5.30	1.091
**Q16. Sensitivity and intuition help the Unani Tibb practitioner to provide spiritual care**	5.02	1.229
**Q17. Being with a patient is a form of spiritual care**	4.86	1.069
**Q18. Unani Tibb practitioners provide spiritual care by respecting the religious and cultural beliefs of patients**	5.09	1.096
**Q19. Unani Tibb practitioners provide spiritual care by giving patients time to discuss and explore their fears, anxieties and troubles**	5.00	1.181
**Q26. Unani Tibb practitioners provide spiritual care by respecting the dignity of patients**	5.20	0.878
**Factor 4—Attitudes to Spiritual Care**	5.05	0.578
**Q21. Spiritual care enables the patient to find meaning and purpose in their illness**	5.36	0.613
**Q22. Spiritual care includes support to help patients observe their religious beliefs**	5.11	0.868
**Q24. I am comfortable providing spiritual care to patients**	4.84	0.914
**Q31. Spiritual care should be instilled throughout the Unani Tibb practitioner’s education programme**	5.11	0.722
**Q32. Spiritual care should be positively reinforced in Unani Tibb practice**	5.23	0.711
**Q40. A team approach is important for spiritual care**	4.43	1.301
**Q35. Spiritual care is important because it gives patient hope**	5.30	0.823
**Factor 5—Spiritual Values**	5.08	0.653
**Q9. Without spirituality, a person is not considered whole**	5.05	0.861
**Q10. Spiritual needs are met by connecting oneself with other people, higher power or nature**	5.23	1.031
**Q11. Spiritual care is an integral component of holistic Unani Tibb therapy**	5.20	1.091
**Q12. Spiritual care is more than religious care**	5.14	0.979
**Q13. Unani Tibb therapy, when performed well, is itself, spiritual care**	4.82	1.147

Range for each item is from 1 (strongly disagree) to 6 (strongly agree), the higher the scores, the higher the agreement

The items with the highest mean value among the five factors include: (1) ‘Attributes for Spiritual Care’ showed practitioners’ concurred that spiritual care requires awareness of one's spirituality (2) ‘Spirituality Perspective’ the maximum mean value indicated that practitioners’ agreed that spirituality is an important aspect of human beings, (3) ‘ Defining Spiritual Care’ the uppermost mean score was for the item which views spiritual care as a process and not a one-time event or activity (4) regarding factor 4, the uppermost score was recorded for practitioners’ belief that spiritual care facilitates meaning and purpose for patients during their illness. The maximum score in factor 5 demonstrated that participants believed that spiritual needs are met by connecting oneself with other people, a higher power or nature. The SCGS indicated good reliability with a Cronbach’s alpha of 0.91 for this sample of Unani Tibb practitioners.

### Responses to the SSCRS

Table 3 provides responses to the SSCRS. This study revealed that the lowest mean scores found were for item 4 ‘I believe spirituality involves only going to Church/Place of Worship’ (1.80; SD = 0.995). The item with the uppermost mean score was calculated for item 14 ‘I believe Unani Tibb practitioners provide spiritual care by respecting the privacy, dignity and religious/cultural beliefs of patients’ (4.52; SD = 0.628). Following descriptive analysis, factors’ mean scores were calculated to obtain the results. The four factors include: (1) Spirituality (M = 3.85; SD = 0.405); (2) Spiritual Care (M = 4.18; SD = 0.603); (3) Religiosity (M = 2.85; SD = 0.691) and (4) Personalised Care (M = 4.16; SD = 0.616).

**Table 3:** Scores on the SSCRS (*n* = 44)

Variables	*M*	*SD*
**Factor 1—Spirituality**	3.85	0.405
**Q6. I believe spirituality is about finding meaning in the good and based on events of life**	4.14	0.702
**Q8. I believe Unani Tibb practitioners can provide spiritual care by enabling a patient to find meaning and purpose in their illness**	4.34	0.745
**Q9. I believe spirituality is about having a sense of hope in life**	4.39	0.538
**Q10. I believe spirituality is to do with the way one conducts one’s life here and now**	4.23	0.803
**Q12. I believe spirituality does not include areas such as art, creativity and self-expression**	2.18	0.995
**Factor 2—Spiritual Care**	4.18	0.603
**Q1. I believe Unani Tibb practitioners can provide spiritual care by arranging a visit by the hospital Chaplain or the patient’s own religious leader if requested**	3.48	1.303
**Q2. I believe Unani Tibb practitioners can provide spiritual care by showing kindness, concern and cheerfulness when giving care**	4.25	0.991
**Q7. I believe Unani Tibb practitioners can provide spiritual care by spending time with a patient giving support and reassurance especially in time of need**	4.25	0.839
**Q11. I believe spirituality is a unifying force which enables one to be at peace with oneself and the world**	4.41	0.726
**Q14. I believe Unani Tibb practitioners can provide spiritual care by having respect of privacy, dignity and religious and cultural beliefs of patient**	4.52	0.628
**Factor 3—Religiosity**	2.85	0.691
**Q4. I believe spirituality involves only going to Church/Place of Worship**	1.80	1.193
**Q13. I believe Unani Tibb practitioners can provide spiritual care by listening to and allowing patients’ time to discuss and explore their fears, anxieties and troubles**	4.27	0.949
**Q16. I believe spirituality does not apply to Atheists or Agonists**	2.50	1.000
**Factor 4—Personalised Care**	4.16	0.616
**Q14. I believe Unani Tibb practitioners can provide spiritual care by having respect of privacy, dignity and religious and cultural beliefs of patient**	4.52	0.628
**Q15. I believe spirituality involves personal friendship and relationships**	3.77	1.054
**Q17. I believe spirituality include people’s morals**	4.20	0.701

Range for each item is from 1 (strongly disagree) to 5 (strongly agree), the higher the scores, the higher the agreement

The scores showed consistent consensus among the practitioners regarding their belief that spirituality is a unifying force, which empowers one to be at peace with oneself and the world. Participants believed that spirituality includes having a sense of hope in life. It was evident in this study that participants believed that Unani Tibb practitioners can provide spiritual care by empowering patients to find meaning and purpose in their illness.

Two items had a mean score of 4.25: item 2 indicated participants agreed that Unani Tibb practitioners could provide spiritual care by displaying kindness, concern and cheerfulness in practice. Item 7 revealed that the participants believed that Unani Tibb practitioners can offer spiritual care by spending time with a patient providing support and reassurance. The results further confirmed that participants believed that Unani Tibb practitioners could provide spiritual care by respecting the privacy, dignity, religious and cultural rights of patients.

### Responses on the SUTS

The scores obtained from the SUTS calculations are presented in Table 4. The results indicated the lowest mean score of 2.86 (SD = 0.995) in item 11 ‘I use spiritual assessments to evaluate my client’s spiritual needs’ and highest score of 4.50 (SD = 0.762) in ‘Spirituality is an integral part of the human experience’. Regarding the factors of SUTS, this study presents the mean score: (1) spirituality in the scope of practice (3.85; SD = 0.448), (2) formal education and training in spirituality (3.52; SD = 0.503) and (3) a need for future educational opportunities and training to address spirituality (4.33; SD = 0.513).

**Table 4:** Scores on the SUTS (*n* = 44)

Variables	*M*	*SD*
**Factor 1—Spirituality Scope of Practice**	3.85	0.448
**Q1. My formal education has adequately prepared me to address my clients’ spiritual needs**	3.00	1.121
**Q2. My treatment sessions would be enhanced if I had more education about how to address my clients’ spiritual needs**	4.16	0.713
**Q3. I would like to pursue further education about how to address my clients’ spiritual needs**	4.02	0.762
**Q4. I try to find more information on spirituality as it relates to Unani Tibb practice**	3.77	0.831
**Q5. I would benefit from attending an educational workshop about addressing and evaluating the spiritual needs of my clients**	4.30	0.553
**Factor 2—Spirituality in Education and Training**	3.52	0.503
** Q6. Spirituality should be addressed by Unani Tibb practitioners**	4.07	0.818
** Q7. It is the client’s responsibility to inform the Unani Tibb practitioner of their spiritual needs**	3.00	1.818
** Q8. My experience as an Unani Tibb practitioner has prepared me to adequately address my client’s spiritual needs**	3.34	0.834
** Q9. I feel comfortable addressing spirituality with my clients**	3.73	1.086
** Q10. It is my responsibility to address my client’s spiritual needs**	3.50	1.045
** Q11. I use spiritual assessments to evaluate my client’s spiritual needs**	2.86	0.950
** Q12. I am aware of various assessments that address spiritual needs of my clients**	2.93	1.021
** Q13. I am confident addressing the spiritual needs of my clients when their beliefs are similar to my own**	3.91	0.960
** Q14. I believe that treating my client’s spiritual need has a direct effect on my client’s quality of life**	4.25	0.890
** Q15. I treat my client’s spiritual needs**	3.61	0.945
**Factor 3—Need for Future Educational Opportunities and Training to Address Spirituality**	4.33	0.513
**Q16. Spirituality helps clients define their therapeutic goals**	4.20	0.660
**Q17. Spirituality helps clients define who they are**	4.30	0.594
**Q18. Spirituality is an integral part of the human experience**	4.50	0.762

Range for each item is from 1 (strongly disagree) to 5 (strongly agree), the higher the scores, the higher the agreement

Regarding the results for factor 1 the inclusion of spirituality in the scope of practice, the participants agreed that educational workshops about addressing and evaluating the spiritual needs of clients would be of benefit to them. The lowest mean score for factor 1 was found for item 1 ‘My formal education has adequately prepared me to address my clients’ spiritual needs’. Additionally, participants agreed that spirituality helps clients define who they are. Furthermore, participants agreed that treating their client’s spiritual needs would directly benefit their client’s quality of life.

## Discussion

Topics on spirituality and spiritual care in the training and practice of Unani Tibb are lacking at a national and international level [1, 2, 14, 15]. Added to this is the evident scarcity of research regarding Unani Tibb practitioners’ perceptions on spirituality and spiritual care in clinical practice. Studies conducted on the Unani Tibb profession in other countries, have mainly focussed on the spiritual personality of participants [14, 15, 26]. This work represents the only study conducted in South Africa that investigated Unani Tibb practitioners' perspectives about spirituality and spiritual care.

Overall, participating Unani Tibb practitioners scored high on the SCGS when compared to previous studies in other health professions indicating a heightened level of spiritual awareness for this cohort [18, 22, 27–35]. This is an unexpected finding in light of the paucity of spirituality related topics in the training, practice and research of Unani Tibb in South Africa. A possible reason for this finding could be attributed to the individualised patient care approach in Unani Tibb which is based on the concept of temperament. Temperament is the combination of a person’s unique physical, mental, emotional and spiritual attributes. This is the reference point to diagnosis and treatment in Unani Tibb [1–3, 25]. The knowledge of temperament may have contributed to a heightened spiritual awareness in the clinical setting.

The lack of spirituality and spiritual care content in the training and practice of other modalities of CAM is a common finding despite that the literature confirms that many people seek CAM treatments for the spiritual component. However, in other health professions such as nursing, occupational therapy and social work these topics has gained attention over the past few decades both nationally and internationally.

In this study, Unani Tibb practitioners agreed that spirituality is an important aspect of human beings. They concurred that spiritual care requires awareness of one's spirituality. These findings agree with previous studies [5, 10, 12, 17, 18, 35–39] which reported that CAM practitioners recognised the integral role of spirituality in the lives of their patients. CAM practitioners also reported that their inclusion of spiritual care into treatment was based on their personal views of spirituality [14, 15, 31–35]. In this study, the spiritual perspectives of Unani Tibb practitioners appeared to be humanistic in nature. Unani Tibb focuses on the science of medicine and the art of care [1, 2]. The art of care refers to the holistic nature of the profession. In addition, practitioner’s spiritual or religious affiliations may have also contributed to their humanistic perspectives.

### Spiritual care attributes

Regarding factor 1 on the SCGS, practitioners expressed agreement about the humanistic attributes such as spiritual awareness, coping, empathy, and establishing trust required for spiritual care. In this study the majority of practitioners regarded themselves as spiritual which may have contributed to this result. This finding was supported by Ahmad [26], which reported that spiritual personality scores were positively correlated with emotional empathy by Unani Tibb students. Similar findings were also reported in other healthcare professions [5, 12, 17, 18, 22, 27–37]. The importance of understanding, empathy, trust and good intent by the Unani Tibb practitioner are well established in training and practice. This may have contributed to this outcome.

### Perspectives on spirituality

The participants responses to factor 2 scored high on the SCGS. This result is consistent with findings in occupational therapy [34] and nursing  [5, 18, 21, 30–33, 36]. Unani Tibb practitioners reached consensus regarding conceptualisations of spirituality and spiritual care. For instance, practitioners constantly concurred with the concept of spirituality as (1) characteristic of being human, (2) a unifying force to find meaning and purpose in life, (3) spirituality as part of our inner being, (4) spiritual well-being is important for one’s emotional well-being, and (5) spirituality drives individuals to search for answers about meaning and purpose in life.

These results suggest a high level of spiritual awareness by this sample of middle aged (median age was 38.4 years) Unani Tibb practitioners from various ethno-cultural backgrounds. Similar findings were reported for studies conducted in other regions [5, 14, 15, 18, 21, 30–33, 36].

### Defining spiritual care

Factor 3 revealed that practitioners perceived spiritual care as a process and not a once-off activity or event. This finding reveals that practitioners perceive that they need to respect patients’ religious beliefs, personal beliefs, cultural beliefs and dignity. This result is consistent with Mthembu et al. [34], who reported similar findings for occupational therapy students in the South African context. Previous studies in other regions reported similar findings for the nursing profession [5, 18, 21, 30–33, 36]. The role of multicultural competence has gained importance over the past few years in the training and practice of health professionals in the South African context. This may have contributed to this finding for Unani Tibb practitioners.

Even though the assessment of spiritual needs is an integral component of spiritual care, Unani Tibb practitioners did not perceive this. Item 17 being with the patient is a form of spiritual care, scored the lowest mean value. This finding agrees with Tiew et al. [30] and Mthembu et al. [34], who reported low mean values for item 17 for student responses in nursing [30] and occupational therapy [34]. Furthermore, these findings have important implications for developing guidelines for spirituality and spiritual care in Unani Tibb clinical practice as these aspects are fundamental to the holistic approach described in Unani Tibb theory [1–3, 14, 15, 25]. The theory–practice gap seems to also exist in the Unani Tibb profession as previously reported in other healthcare professions [5, 10, 13–15, 27, 28, 40].

### Spiritual care attitudes

The overall mean score of factor 4 was low compared to other factors in the SCGS. This finding agrees with Mthembu et al. [34] who reported a low overall mean score for factor 4 when compared to other factors in the SCGS. Unani Tibb practitioners agreed that spiritual care is important because it gives the patient hope and enables the patient to find meaning and purpose in their illness. In addition, practitioners strongly agreed that spiritual care should be positively reinforced in Unani Tibb practice. Similar findings were reported for occupational therapy and the nursing professions [5, 18, 21, 30–34, 36, 40].

The need for spiritual care guidelines for CAM clinical practice has been highlighted in previous studies [10, 14, 15, 37–39]. Moreover, according to the WHO Benchmarks for the Practice and Training in Unani Medicine (Tibb) the recommended training programmes do not include any modules or topics on spirituality and spiritual care [1, 2, 14, 15]. This further supports the need for the development of guidelines for spirituality and spiritual care in Unani Tibb clinical practice.

Item 24 on the SCGS (I am comfortable providing spiritual care to patients) scored a low mean value. This finding agrees with previous studies which reported on the barriers to spiritual care for CAM practitioners which included ‘insufficient knowledge /training’, ‘insufficient time’, ‘general discomfort’, and a ‘concern of offending the patients’ [14, 15, 20, 39, 41]. Unani Tibb practitioners in South Africa may feel unprepared in providing spiritual care due to insufficient knowledge and general discomfort however further studies are needed to confirm this. Spirituality and spiritual care topics are lacking in Unani Tibb textbooks, curriculum and clinical documents. This suggests that Unani Tibb practitioners in South Africa are not adequately prepared to deliver spiritual care.

In this study the lowest mean score recorded for factor 4 was for item 40 (a team approach is important for spiritual care). This result is consistent with the findings of past studies by Tiew [30] and Mthembu [34] which recommended a multi-disciplinary approach towards addressing the spiritual needs of patients. Spiritual care is based on a multi-disciplinary team approach which begins with patient evaluation followed up by appropriate strategies to address spirituality [5, 42].

### Spiritual care values

Responses by practitioners to factor 5 scored the least overall mean score on the SCGS. However, practitioners constantly concurred on item 10 (spiritual needs require connecting oneself with others, nature or a higher power) and item 11 (spiritual care is a vital aspect of Unani Tibb therapy). These findings agree with other previous studies [5, 18, 22, 36]. In this study the majority of Unani Tibb practitioners reported that they belong to a religion and that they participate in religious activities. This may have contributed to these findings. However, less consensus was reached regarding other items. For instance, a low mean score was reported on item 9 (spirituality is needed for a person to be considered whole). These results agreed with previous studies [30–34].

The lowest score was on item 13 which stated that “Unani Tibb therapy, when performed well, is itself, spiritual care.” This suggests that Unani Tibb practitioners may not feel adequately equipped to address the spiritual needs of patients. Other studies also reported that healthcare practitioners felt ill-prepared to address the subject of spirituality with their patients [5, 7, 8, 13–15, 18, 34, 36, 43]. Furthermore, ‘insufficient knowledge / training’ was cited as one of several barriers to spiritual care for CAM practitioners reported in previous studies [14, 15, 20, 39, 41].

In this study, practitioner responses had high overall mean scores on the SCGS. These findings suggest that Unani Tibb practitioners were spiritually aware and are keen to provide spiritual care in the clinical setting. Similar results were recorded in other studies within CAM professions [7, 8, 12–14, 41].

### Attitudes towards spirituality

For factor 1 on the SSCRS, participants highly agreed that Unani Tibb practitioners can provide spiritual care by empowering a patient to find meaning and purpose in their illness. Similarly, practitioners also agreed that spirituality is about having hope in life and that spirituality includes the way one lives one’s life in the present. These findings concur with the holistic approach described within Unani Tibb theoretical principles. In Unani Tibb philosophy diseases are multi-dimensional, which includes the physical, emotional, mental, and spiritual components [1–3, 14, 15, 24, 25, 44].

The human body comprises of a physical body and spirit. These two entities are inseparable and good health is maintained when there is harmony between the physical and spiritual functions [3, 14, 15, 24, 25]. Furthermore, these findings agree with other studies that reported that humans have basic requirements which includes self-worth, purpose, meaning, choice, control and occupation [5, 18, 22, 34, 36].

### Spiritual care perspectives

Regarding factor 2 practitioners expressed the highest agreement on item 14 (I believe Unani Tibb practitioners can provide spiritual care by having respect for the privacy, dignity and religious and cultural beliefs of patients). Followed by item 11 (I believe spirituality is a unifying force, which enables one to be at peace with oneself and the world). Practitioners also equally agreed on two items: “I believe Unani Tibb practitioners can provide spiritual care by showing kindness, concern and cheerfulness when giving care” and “I believe Unani Tibb practitioners can provide spiritual care by spending time with a patient giving support and reassurance especially in time of need.”

A possible explanation for these findings may be found in the understanding of medical ethics in Unani Tibb. Medical ethics within Unani Tibb are derived from the teachings of three historic pioneers of medicine: Hippocrates, Galen and Ibn Sina [1–3, 14, 15, 24, 25, 45].

Ibn Sina describes ethical subjects within three main domains: considering patient interests, communication skills, and observing the characteristics of professional excellence [45]. The findings are consistent with those of Mthembu et al. [34] who cited the understanding of ethical principles such as autonomy, beneficence, justice and non-maleficence as a reason to the responses of occupational students to factor 2 on the SSCRS. Similar findings were also reported in the nursing profession [5, 18, 21, 36].

### Religiosity

Regarding factor 3 on the SSCRS, practitioners expressed the highest agreement on item 13 (I believe Unani Tibb practitioners can provide spiritual care by listening to and allowing patients’ time to discuss and explore their fears, anxieties and troubles). A possible explanation for the above findings may be found in the recommended counselling methods described by Ibn Sina which forms part of the guiding principles for the Unani Tibb profession. Concerning communication skills, Ibn Sina advises: *“… upon visiting a patient, the physician should sit next to him in a way that is in front of him so that he could see his face and listen to him well. He should only ask the questions that provide necessary information for his diagnosis and treatment: the physician ought to avoid extraneous and unnecessary questions. Moreover, the physician should avoid prolonging the time of his visit even if the patient wants his companion. The physician should limit the visiting time to the required length in a compassionate and respectful manner”* [45].

Regarding Ibn Sina’s view on how to increase the patient’s hope during times of distress: *“The physician should also be calm and dignified in his behaviour, associated with patience, gentleness and tolerance; he must listen well to the patient’s complaints, explain the information he needs according to the patient’s understanding, and avoid using difficult words in the conversation with the patient”* [45]*.* Similar findings were reported in other studies [5, 21, 22, 34, 36].

### Personalised care

Regarding factor 4, practitioners expressed the highest agreement on item 14 (I believe Unani Tibb practitioners can provide spiritual care by having respect of privacy, dignity and religious and cultural beliefs of patient). Followed by item 17 (I believe spirituality includes people’s morals). In the current study, the concept of spirituality includes morals which concurs with the findings of Kızılca Çakaloz et al. [5], McSherry et al. [22], Mthembu et al. [34], Herlianita et al. [36] and McSherry et al. [46]. The South African population is diverse, for this reason multicultural competence has become a vital component in the training and practice of Unani Tibb. This may account for the findings in this study.

### Spirituality in the scope of practice

In this study, practitioners reported the lowest overall mean value on the SUTS scale compared with SCGS and SSCRS. Regarding factor 1, the participants reported the highest agreement for item 6 (spirituality should be addressed by Unani Tibb practitioners) and they expressed less agreement on item 12 (I am aware of various assessments that address spiritual needs of my clients). They also scored high in item 18 (spirituality is an integral part of the human experience). These results corroborate the findings of previous studies which reported that most CAM practitioners view spirituality as an important aspect of a human being’s life [7–12].

These findings are also supported by previous studies which reported that CAM practitioners valued the role of spirituality as a component of holistic care. However, they cited a lack of knowledge as one of the barriers to achieving a holistic practice [14, 15, 39, 41].

### Spirituality in education and training

In factor 2 (education) and factor 3 (need for future educational opportunities and training to address spirituality) on the SUT scale, practitioners indicated less agreement about their formal education adequately preparing them to address the spiritual needs of their clients. Practitioners also expressed the highest agreement on item 5 (I would benefit from attending an educational workshop about addressing and evaluating the spiritual needs of my clients). These results corroborate the findings of previous studies on CAM and Unani Tibb. CAM practitioners are reported to be ill equipped to address the topic of spirituality with their patients [7, 8, 13–15, 43]. These findings also concur with previous studies in the occupational therapy and nursing professions [5, 18, 21, 30–33, 36].

## Limitations of the study

The findings of this study should be considered in the light of several limitations. This study only included Unani Tibb practitioners from one region (South Africa). Therefore, the findings may not be generalised. The small sample size of this study was another limitation. This study was explorative as the Unani Tibb field is a specialised one in the South African context with a low number of registered practitioners (n = 68) and a lack of research on this population. This research work used a cross-sectional study design which has limitations as it does not provide the causal associations among the study’s variables. A convenience sample method was used in this study.

Participants who volunteered in this study may have a specific interest in this topic. Their views may differ from other practitioners who were not part of this study. The use of an online self-reported questionnaire may have influenced how the participants responded and may have led to overestimation. A quantitative research design was used in this study, a qualitative approach may provide a more complete and clearer picture of the phenomenon under investigation.

## Conclusion

The key findings of this study indicate that Unani Tibb practitioners have a high regard for spirituality and spiritual care despite the lack of formal training on these topics. This study also reveal that Unani Tibb practitioners are unprepared to address aspects of spirituality and spiritual care in clinical practice. This compromises the holistic approach required by the profession.

Consequently, this study highlighted the need for guidelines on spirituality and spiritual care to improve holistic care in the Unani Tibb profession in South Africa. For this to occur future studies should explore Unani Tibb practitioners’ perceptions regarding spirituality and spiritual care through qualitative research to provide a much deeper understanding to this phenomenon.

## Data Availability

The datasets used and/or analysed during the current study are available from the corresponding author on reasonable request.

## Abbreviations

CAM: Complementary and Alternative Medicine
WHO: World Health Organization
SCGS: Spiritual Care-Giving Scale
SSCRS: Spirituality and Spiritual Care Rating Scale
SUTS: Spirituality in Unani Tibb Scale
SOT: Spirituality in Occupational Therapy
SATA: South African Tibb Association
SPSS: Statistical Package for the Social Sciences
NRF: National Research Foundation of South Africa
